# Autonomic arousal in childhood anxiety disorders: Associations with state anxiety and social anxiety disorder

**DOI:** 10.1016/j.jad.2014.11.056

**Published:** 2015-04-01

**Authors:** Anna Alkozei, Cathy Creswell, Peter J. Cooper, John J.B. Allen

**Affiliations:** a University of Reading, United Kingdom; b Stellenbosch University, South Africa; c University of Arizona, United States

**Keywords:** Social anxiety disorder, Childhood anxiety disorders, Autonomic flexibility, Respiratory sinus arrhythmia, Heart rate

## Abstract

**Background:**

Psychophysiological theories suggest that individuals with anxiety disorders may evidence inflexibility in their autonomic activity at rest and when responding to stressors. In addition, theories of social anxiety disorder, in particular, highlight the importance of physical symptoms. Research on autonomic activity in childhood (social) anxiety disorders, however, is scarce and has produced inconsistent findings, possibly because of methodological limitations.

**Method:**

The present study aimed to account for limitations of previous studies and measured respiratory sinus arrhythmia (RSA) and heart rate (HR) using Actiheart heart rate monitors and software (Version 4) during rest and in response to a social and a non-social stressor in 60 anxious (30 socially anxious and 30 ‘other’ anxious), and 30 nonanxious sex-and age-matched 7–12 year olds. In addition, the effect of state anxiety during the tasks was explored.

**Results:**

No group differences at rest or in response to stress were found. Importantly, however, with increases in state anxiety, all children, regardless of their anxiety diagnoses showed less autonomic responding (i.e., less change in HR and RSA from baseline in response to task) and took longer to recover once the stressor had passed.

**Limitations:**

This study focused primarily on parasympathetic arousal and lacked measures of sympathetic arousal.

**Conclusion:**

The findings suggest that childhood anxiety disorders may not be characterized by inflexible autonomic responding, and that previous findings to the contrary may have been the result of differences in subjective anxiety between anxious and nonanxious groups during the tasks, rather than a function of chronic autonomic dysregulation.

## 1 Introduction

Anxiety disorders, and social anxiety disorder in particular, are common in childhood, are frequently chronic if left untreated, and are associated with emotional distress as well as impairment in social and academic functioning (Essau et al., 2000; Ezpeleta et al., 2001; Mychailyszyn et al., 2010; Newman et al., 1996). Even though childhood anxiety can be treated effectively through the use of Cognitive Behavior Therapy (CBT), in approximately 40% of cases CBT does not lead to a substantial reduction of anxiety symptoms (In-Albon and Schneider, 2007) and, notably, the presence of social anxiety disorder has been found to be associated with especially poor treatment outcomes from generic treatments (Hudson et al., 2010). In order to improve treatment outcomes for anxious children, particularly those with social anxiety disorder, a better understanding of potential maintaining processes is required.

Psychophysiological theories suggest that anxiety disorders may be associated with chronic dysregulation of the autonomic nervous system. Friedman׳s (2007) autonomic flexibility-neurovisceral integration model proposes that individuals with anxiety disorders evidence inflexibility in their autonomic response both in the absence of stress and when responding to stressors. In relation to activity of the parasympathetic nervous system in particular, the model predicts that, in comparison to nonanxious populations, individuals with anxiety disorders will (i) display increased heart rate (HR) and diminished respiratory sinus arrhythmia (RSA) (respiratory linked heart-rate variability (HRV)) at baseline, (ii) fail to substantially increase HR and decrease RSA in response to a stressor, and (iii) recover more slowly once the stressor has passed.

Studies investigating the role of autonomic parasympathetic arousal in childhood anxiety disorders have produced conflicting findings, with some studies showing higher HR and/or lower RSA at baseline in children and adolescents with anxiety disorders when compared to controls (Henje Blom et al., 2010; Monk et al., 2001; Sharma et al., 2011a) and others failing to show such differences (Kossowsky et al., 2012; Kristensen et al., 2014; Sharma et al., 2011b; Yeragani et al., 2001). In addition, while one study has found lower reactivity to a stressor (i.e., less change from baseline in response to a task) in anxious groups in comparison to control groups (Monk et al., 2001), another study has shown greater reactivity (Kossowsky et al., 2012), and some studies have found no differences (Beidel, 1991; Kristensen et al., 2014; Sharma et al., 2011b). Interpreting these mixed findings is difficult because of a number of methodological factors. Specifically children participating in these studies have typically been drawn from a broad age range (6–18 years) (Monk et al., 2001; Sharma et al., 2011a, 2011b), anxiety disorder and comparison groups have not been matched on age and gender (Monk et al., 2001), studies have relied on one single stressor only (Kossowsky et al., 2012), or employed physiological rather than psychological stressors (Monk et al., 2001; Sharma et al., 2011b), and/or the effects of respiration have not been taken into account (Beidel, 1991).

The experience of physiological symptoms occupies a central role in models of the maintenance of social anxiety disorder (Clark and Wells, 1995), yet few studies have investigated whether children with social anxiety disorder, specifically, show a different pattern of autonomic arousal compared to children with other anxiety disorders and nonanxious children. Two studies have shown that, compared to nonanxious children, children (8–12 years) with social anxiety disorder had higher HR and lower RSA at baseline, reacted to a social stressor with less change in HR and/or RSA, and took longer to return to their initial baseline levels once the stressor passed (Krämer et al., 2012; Schmitz et al., 2011). This pattern of restrictive autonomic flexibility has also been shown in a group of high socially anxious children in comparison to low socially anxious children drawn from a community population (Schmitz et al., 2013). All of these studies, however, lacked a non-social anxiety disordered comparison group. Thus it remains unclear whether the findings are specific to social anxiety disorder or apply to anxiety disorders in general. In addition, the absence of a non-social stressor makes it difficult to establish whether differences in autonomic activity are only detectable during disorder-specific tasks or whether they generalize to other situations. Furthermore, the socially anxious children in these studies reported elevated levels of anxiety *during* the task compared to nonanxious children, and therefore differences between the groups might have been a reflection of their *current* state rather than *trait* anxiety.

The present study was designed to overcome some of the limitations of previous studies by measuring HR and RSA in children with a primary diagnosis of social anxiety disorder, children with an anxiety disorder other than social anxiety disorder, and healthy control children, with measurements made at baseline and in response to a social and a non-social stress task. The design allowed for the testing of the following specific hypotheses:

1. Children with a current anxiety disorder will display higher HR and lower RSA at baseline in comparison to nonanxious children.
2. Children with a current anxiety disorder will display reduced HR reactivity and RSA reactivity in response to a stressful task in comparison to nonanxious children. This effect will be amplified among socially anxious children in a social stress task, compared to other anxious and nonanxious children.
3. Children with a current anxiety disorder will display reduced HR recovery and RSA recovery after a stressful task in comparison to nonanxious children. This effect will be amplified among socially anxious children in a social stress task, compared to other anxious and nonanxious children.

In addition, the impact of differences in children׳s state anxiety during the tasks on differences in autonomic activity was examined in response to and during recovery from stressful tasks.

## 2 Method

### 2.1 Participants

Ninety children aged 7–12 years took part in the study. Thirty children met diagnostic criteria for a primary diagnosis of social anxiety disorder (SA), 30 met diagnostic criteria for an anxiety disorder but not social anxiety disorder (ANX), and 30 were selected on the basis of having anxiety levels within a non-clinical range (NONANX). The number of male (*n*=14) and female (*n*=16) participants in each group was the same and groups were of similar age (*F*(2, 89)=.03, *p*=.96) (see Table 1).

Children in the clinical groups were by referred to the Berkshire Child Anxiety Clinic (BCAC) at the University of Reading by local health and education service personnel as part of a larger investigation. In addition to the specified age, the other requirement for inclusion was that they meet criteria for a current primary anxiety disorder diagnosis. Exclusion criteria were (a) significant physical or intellectual impairment (where this would impede reliable completion of measures), (b) current prescription of psychotropic medication (or if on medication this should have been stable for a month), however none of the participants was prescribed psychotropic medication at the time of the assessment; and (c) previous receipt of six or more sessions of cognitive behavior therapy (i.e., treatment specifically targeting the processes under investigation by the larger study). Following referral, children and their primary caregiver were invited for an initial clinical assessment where they were interviewed about their child׳s anxiety disorder using the Anxiety Disorder Interview Schedule (ADIS-c/p, see below).

NONANX participants were volunteers, recruited through invitation letters, sent predominantly through schools and local after-school clubs, specifically asking for children to form a non-anxious comparison group. Children were screened on the basis of child and parent report on the Spence Children׳s Anxiety Scale-child and parent versions (SCAS-c/p; see below). The inclusion criteria were that children must be within 7–12 years and have anxiety levels within the normal range, based on both parent and child report (SCAS-c/p; see below). Families in the NONANX condition were given gift tokens in exchange for taking part.

Children in the SA group, by definition, all had a primary diagnosis of social anxiety disorder. In addition, 80% met criteria for a secondary anxiety disorder. Children in the ANX group had a primary diagnosis of an anxiety disorder other than social anxiety disorder. Table 2 summarizes children׳s primary and comorbid anxiety, and their depressive and externalizing diagnoses.

### 2.2 Psychometric measures

#### 2.2.1 Structured diagnostic interview with children and parents

Children were assigned diagnoses on the basis of the Anxiety Disorders Interview Schedule for DSM IV for Children- Child and Parent Versions (ADIS-C/P; Silverman and Albano, 1996). Where children met symptom criteria for a diagnosis (based on either child or parent report) they were assigned a clinical severity rating (CSR) ranging from 0 (absence of psychopathology) to 8 (severe psychopathology). Only those children who met symptom criteria with a CSR of 4 or more were considered to meet diagnostic criteria. Assessors were trained on the administration and scoring of the ADIS-C/P through verbal instruction, listening to assessment audio-recordings and participating in diagnostic consensus discussions. The first twenty ADIS-child and ADIS-parent interviews conducted were then discussed with a consensus team, led by an experienced diagnostician (Consultant Clinical Psychologist). The assessor and the consensus team independently allocated diagnoses and CSRs. Following the administration of 20 interviews, inter-rater reliability was checked. Overall reliability for the team was excellent. Reliability for presence or absence of each separate diagnosis was kappa =.98; and for the CSR intra-class correlation=.99.

#### 2.2.2 Symptoms of anxiety

The Spence Children׳s Anxiety Scale (SCAS-c/p; Spence, 1998) was administered to assess child and parent reported symptoms of anxiety. The child version is a self-report questionnaire that requires children to rate how often they experience each of the 38 anxiety symptoms, presented alongside six positive filler items, on a 4 point scale from ‘never’ (0) to ‘always’ (3). The SCAS has demonstrated high internal-consistency reliability and concurrent validity (Spence, 1998), with children from 7 years of age. Internal consistency was good (*α*=.90 for SCAS-c, *α*=.94 for SCAS-p). In the present study the social phobia subscale, consisting of 6 items, was used as an indicator of self and parent reported social anxiety symptoms. Internal consistency for the social phobia subscale was acceptable (*α*=.74 for SCAS-c, *α*=.87 for SCAS-p).

### 2.3 Cardiovascular measures

Cardiovascular activity was measured using Actiheart monitors and software (Cambridge Neurotechnology, Cambride, UK). Actiheart monitors were attached to the child׳s chest using 2 standard ECG electrodes. Electrodes were placed below the sternum and from this position horizontally towards the left side of the body. Actiheart monitors calculate Interbeat Interval (IBI) data by detecting R waves of the ECG, and recording the time between them. After the recording, the data were transferred to the Actiheart software for IBI inspection and editing. The semi-automated editing program of the Actiheart software detects artifacts in the IBI series and corrects them. This was followed by manual inspection of the complete IBI series and the correction of remaining artifacts. HRV in the HF band (.12–.4 Hz), which is assumed to represent vagal influences, was derived with CMetX Cardiac Metric Software (Allen et al., 2007) and used to calculate an estimate of RSA. The CMetX program converted the IBI series to a time-series sampled at 10 Hz with linear interpolation. A 241-point optimal finite impulse response digital filter designed using FWTGEN V3.8 (Cook and Miller, 1992) with half-amplitude bandpass frequencies of .12–.40 Hz was applied to the 10 Hz time-series representation of the IBI series. The natural log of the variance of the filtered waveform was used as the estimate of RSA. To ensure that participants׳ breathing rate did not influence RSA, (e.g., breathing outside the .12–.40 Hz band), the dominant frequency in the power spectrum of the respiration waveform was examined for each participant. Analyses were rerun excluding participants who did not breathe within the assigned breathing range, remained consistent throughout and are therefore not reported below. Simultaneously, an internal uniaxial accelerometer sensed the frequency and intensity of the subject׳s torso movements at magnitudes ranging from .05–2.00 G and a frequency response from .25–2.5 Hz, and recorded them as movement counts every 15 s. This variable has been used as a measure of physical activity level during the tasks, in order to control for any autonomic changes based on movement.

### 2.4 Procedure

On arrival for the lab assessment, children and their mothers were given 5 min to acclimate to the laboratory playing a familiar game (‘Connect Four’). After this the Actiheart monitors were placed on the child and mother. The child was then taken to a neighboring room to complete a battery of questionnaires for approximately 45 min (not part of the current investigation). After this the child was asked to choose a DVD to watch. Children׳s weight (kg) and height (m) was recorded, as this might have influenced their autonomic activity (Silvetti et al., 2001). Mother and child, seated separately, watched the DVD for 5 min (*Baseline*). Following this, they were told that the child would be asked to give a presentation to the researcher, which would be recorded on the video camera. Children were given a choice of topics to talk about (e.g., ‘My hobbies’) and were told that they would be left for 5 min to prepare and then would be asked to give the speech to the researcher and camera for 3 min (*Social Stress Task*), following the procedure described in Creswell et al. (2013). After the task a measure of state anxiety was obtained whereby children were asked how scared they felt while doing the task (0=not scared at all, 10=very scared). The child and mother continued to watch the DVD for another 5 min (*Baseline 2*) after which they were presented with a difficult puzzle asking them to put smaller shapes together to form one larger shape, following the procedure by Hudson and Rapee (2001), designed in such a way that it was unlikely that the child was able to complete the task within the 5 min time limit. If children were able to complete the puzzle within 5 min, they were immediately given a new, more difficult, puzzle to complete (*Cognitive Stress Task*). After the task, children were asked again how scared they felt during the task and continued to watch the DVD for another 5 min (*Baseline 3*).

### 2.5 Ethical considerations

This study was reviewed by the Local Research Ethics Committee on behalf of the National Health Service and the University of Reading Research Ethics Committee. Mothers and children were both provided with written and verbal information about the study. In order to participate in the study, written maternal consent and child assent were both required.

## 3 Results

### 3.1 Data analysis

The data were analyzed using IBM SPSS Statistics V.20 using separate multivariate analyses of variance (MANOVA) to investigate the baseline differences in RSA and HR, as well as RSA and HR reactivity to and recovery from the social stress and the cognitive stress task. Analyses were considered statistically significant at *p*<.05 (two-sided). Missing data were present for some of the questionnaires; as a result degrees of freedom vary slightly from analysis to analysis. Data for all physiological variables were present for the complete sample. No statistical outliers were found. Cohen׳s *d* was used as a measure of effect size.

### 3.2 Sample characteristics

Children in the SA group had a significantly greater number of diagnoses compared to children in the ANX group (*t*(58)=2.85, *p*=.006). This reflects the fact that the groups have similar profiles on anxiety disorders other than social anxiety disorder (mean number of non-SA diagnoses for the SA and ANX groups, *M*=1.86 and 1.93 respectively). The rates of mood disorders did not differ between the two anxious groups (*χ*²(1)=.22, *p*=.64), however, children in the SA group had a somewhat higher frequency of behavioral disorders than children in the ANX group (*χ*²(1)=2.78 *p*=.09). Analyses were rerun excluding children with a behavioral disorder. Results remained consistent throughout and are therefore not reported below.

Analyses were conducted to confirm group differences on total and social anxiety symptoms. As expected, significant group differences were found on total anxiety (SCAS-c, *F*(2, 86)=7.96, *p* <.001; SCAS-p, *F*(2, 82)=36.75, *p*<.001) and social anxiety symptom scores (SCAS-c social anxiety, *F*(2, 81)=6.88, *p* <.01; SCAS-p social anxiety, *F*(2, 80)=10.70, *p*<.001). As shown in Table 1, for total SCAS-p score post-hoc tests identified significant differences between both clinical groups (SA and ANX) and the control group (NONANX), as well as between the two clinical groups; and for total SCAS-c scores there was a significant difference between the SA and the NONANX group. There was a significant difference between the SA and both other groups on child and parent report on the social anxiety subscale.

### 3.3 Analyses of possible confounding variables

In order to establish whether groups differed on variables that might have confounded the analyses, group differences on state anxiety, physical activity levels during the tasks, and body mass index were investigated. No differences between the three groups were found for state anxiety ratings for the social stress task (*F*(2, 81)=.48, *p*=.61, *d*=.19) or the cognitive stress task (*F*(2, 81) =.57, *p*=.56, *d*=.05) (see Table 3). In addition, no significant differences in activity levels between the three groups were found for the social stress task (*F*(2, 89) =.72, *p*=.50) or for the cognitive stress task (*F*(2, 88)=1.66, *p*=.19). Furthermore, groups did not differ in body mass index (BMI, kg/m²) (*F*(2, 78) =.99, *p*=.37) (see Table 1).

### 3.4 Hypotheses testing

Hypothesis 1 Hypothesis 1 (i.e., whether there are baseline differences in RSA and HR between the groups) was investigated using a multivariate analysis of variance (MANOVA) for baseline HR and baseline RSA as the dependent variables and group (SA versus ANX versus NONANX) as the independent variable.

**Fig. 1:**
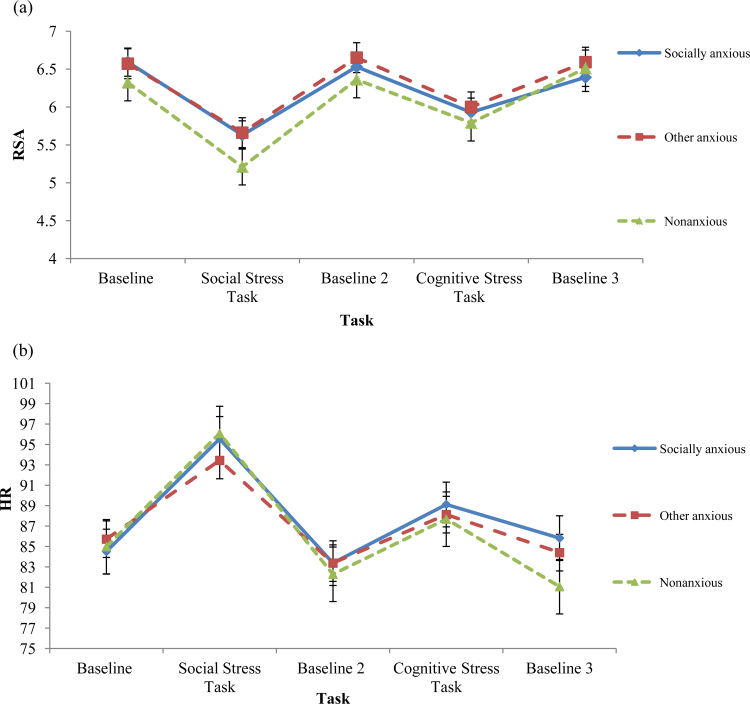
Heart rate (HR) and respiratory sinus arrhythmia (RSA) in response to and during recovery from both tasks. (a) RSA in response to and during recovery from both tasks. (b) HR in response to and during recovery from both tasks.

There was no significant main effect of group on baseline measures of HR and RSA (*F*(2, 86)=1.48, *p*=.12) (see Fig. 1).

Hypotheses 2 and 3 In order to examine hypotheses 2 and 3 (i.e., whether groups differed in RSA and HR reactivity to and recovery from the tasks) and the exploratory question, reactivity and recovery scores were calculated (i.e., *change* scores from pre-task baseline to task and from task to post-task baseline). Reactivity to each of the tasks was described by the following equation: Task – Baseline and recovery from the tasks was described by: Baseline after the task – Task. Two MANCOVAs were conducted for each task separately (one for reactivity scores and one for recovery scores for the social stress task and the cognitive stress task) including the measures of HR reactivity/recovery and RSA reactivity/recovery as dependent variables and group as the independent variable. State anxiety and the group×state anxiety interaction were entered as covariates. State anxiety ratings were centered using the mean as a reference value to overcome colinearity between the group and the group×state anxiety interaction (for the social stress task: unadjusted, *r*(81)=.39, *p*<.001; centered, *r*(81)=.24, *p*=.02; for the cognitive stress task: unadjusted, *r*(82)=.23, *p*=.04; centered, *r*(82)=.23, *p*=.99).

### 3.5 Social stressor

For HR and RSA reactivity to the social stressor, there was no significant effect of group (*F*(2, 84)=1.57, *p*=.21), state anxiety (*F*(2, 83) =.24, *p*=.78), nor a significant group×state anxiety interaction (*F*(2, 84) =.43, *p*=.64) For HR and RSA recovery from the social stressor, there was a marginal effect of group (*F*(2, 84)=2.62, *p*=.08), but no effect of state anxiety (*F*(2, 83)=.60, *p*=.54) and no group×state anxiety interaction (*F*(2, 84)=1.30, *p*=.27). Follow up contrasts showed a marginal association of group and HR recovery (*F*(2, 84)=2.61, *p*=.08, *d*=.42). Table 3 shows that children in the NONANX group had somewhat greater HR recovery scores than children in the ANX group (*k*=6.13, *p*=.02).

### 3.6 Cognitive stressor

**Fig. 2:**
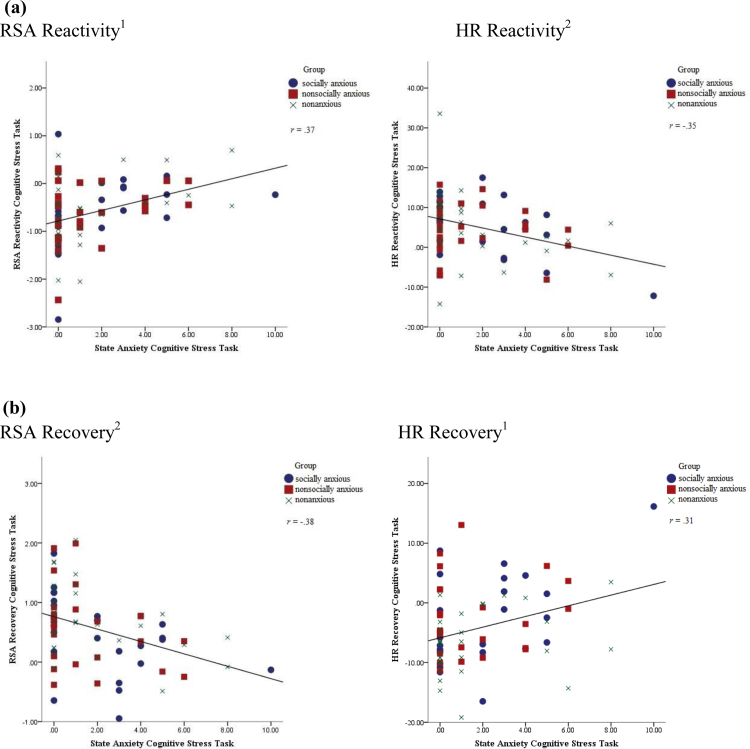
Associations between state anxiety and change in heart rate (HR) and respiratory sinus arrhythmia (RSA) in response to reactivity to and during recovery from the cognitive stress task. ^1^For RSA reactivity and HR recovery, a greater negative score indicates a greater change from baseline to task or vice versa, ^2^For HR reactivity and RSA recovery, a greater positive score indicates a greater change from baseline to task or vice versa. HR and RSA reactivity and recovery scores were calculated as follows: Reactivity=Task HR/RSA – Baseline HR/RSA; Recovery=Baseline after the task HR/RSA – Task HR/RSA.

For HR and RSA reactivity to the cognitive stressor, there was no significant effect of group (*F*(2, 84)=.43, *p*=.64), nor a significant group×state anxiety interaction (*F*(2, 84)=1.62, *p*=.20); however there was a significant effect of state anxiety (*F*(2, 83)=7.94, *p*=.001). Follow up contrasts showed a significant association between state anxiety and both HR and RSA reactivity (*F*(1, 84)=10.06, *p*=.002, *d*=.16 and *F*(1, 84)=12.38, *p*=.001, *d*=.84 respectively). Fig. 2 shows that the more anxiety children reported during the task, the lower their HR and RSA response to the task compared to baseline.

For HR and RSA recovery from the cognitive stressor, as for the social stress task, there was a marginal effect of group, (*F*(2, 84)=2.55, *p*=.08) and also a significant effect of state anxiety (*F*(2, 38)=5.50, *p*=.008); but the group×state interaction was not significant (*F*(2, 84)=1.78, *p*=.17). Follow up contrasts showed a marginal difference in HR recovery between groups, as seen for the social stress task (*F*(2, 84)=2.47, *p*=.09, *d*=.49). Fig. 2 shows that children who reported more anxiety during the task, showed less change in HR and RSA between the task and the subsequent baseline than children who reported less anxiety during the task. In addition, follow up tests showed that there was a significant association between state anxiety and HR and RSA recovery (*F*(1, 84)=7.61, *p*=.007, *d*=.92 and *F*(1, 84)=12.36, *p*=.001, *d*=.93 respectively). In addition, Table 3 shows that children in the NONANX group showed marginally greater HR recovery than both children in the SA and the ANX group (*k*=2.88, *p*=.07 and *k*=3.29, *p* =.04 respectively).

### 3.7 Additional analyses

In order to investigate whether any differences in reactivity and recovery scores were driven by differences at baseline (children who had lower RSA during the baseline condition might not have been able to decrease RSA during the task as much as children who had higher RSA during the baseline condition), two MANOVAs (one for RSA and one for HR) were conducted with HR/RSA during each condition (Baseline, Social Stressor, Baseline 2, Cognitive Stressor and Baseline 3) as the dependent variables and group as the independent variable.

There was a marginal effect of group for HR (*F*(5, 84)=2.30, *p*=.05), however, follow up tests showed no differences in HR between the three groups for any condition (see Fig. 1). In addition, there was a significant effect of group for RSA (*F*(5, 84)=3.53, *p*=.006). Follow up contrasts however, showed that groups differed only marginally during the social stress task (*F*(2, 87)=2.47, *p*=.09, *d*=.49). Children in the NONANX group had marginally lower RSA scores during the social stressor than children in the clinical groups (*k*=.45, *p*=.05 and *k*=.43, *p*=.03 respectively) (see Fig. 1). Analyses including change scores in RSA from baseline in response to the social stressor were rerun accounting for RSA during the social stressor, and remained consistent.

## 4 Discussion

The aims of this study were to investigate autonomic arousal in terms of heart rate (HR) and respiratory sinus arrhythmia (RSA) among children with social anxiety disorder, other anxiety disorders and nonanxious children at rest and in response to general and disorder specific stressors. In addition, the association between state anxiety and children׳s physiological response to stressful tasks were explored. Only limited support for the study hypotheses was found.

Contrary to the first hypothesis, no differences at baseline in HR and RSA between anxious and nonanxious children were found. This stands in contrast to some previous studies (Krämer et al., 2012; Monk et al., 2001; Schmitz et al., 2011; Sharma et al., 2011a), however, many of these studies also reported higher levels of state anxiety in anxious versus nonanxious children (Krämer et al., 2012; Schmitz et al., 2011). Although no measure of state anxiety was obtained during the baseline condition in the present study, there was a long acclimation period (approximately 45 min). Children in the anxious groups might have been more aroused upon arrival, as might have been the case in previous studies, but returned to a resting state once they settled into the lab. The current findings therefore provide no evidence for chronic physiological dysregulation of the parasympathetic nervous system in children with anxiety disorders at rest. This finding is in line with several studies which find no baseline differences in anxious versus nonanxious children (Kossowsky et al., 2012; Kristensen et al., 2014; Sharma et al., 2011b; Yeragani et al., 2001), in contrast to findings from studies with adult and adolescent populations (Henje Blom et al., 2010; Lyonfields et al., 1995). This leads to the intriguing possibility that more prolonged experience of anxiety might lead to changes in autonomic activity at rest. Prospective longitudinal work is needed to examine developmental changes in the association between anxiety and cardiac responses.

No evidence for hypothesis two was found. That is, there were no differences between the groups in terms of HR and RSA reactivity in response to stressful tasks. This might be explained by the fact that, unlike in previous studies, groups in the present study did not statistically differ in terms of subjective anxiety during the task. The findings presented therefore suggest that differences in autonomic activity in response to stress found in previous studies might have been the result of children׳s current emotional state rather than reflective of a dysfunctional autonomic nervous system (Schmitz et al., 2011). It is possible that children with anxiety disorders will more commonly show this autonomic reaction to stress, but there is no evidence to suggest that this response is pathological. It should be acknowledged, however, that, although autonomic arousal was inflated during each stress task in comparison to baseline, children only reported mild to moderate levels of anxiety in response to both stressors. Although the tasks in the present study are commonly used as mild stressors (e.g. Hudson and Rapee, 2001), it is possible that several factors might have led to lower anxiety levels than was anticipated by the present task designs. Possible reasons for this include: (i) children were told that no one would be able to watch the video of their presentation apart from the research assistant; (ii) children׳s mothers remained present during both tasks and were able to help their child when needed; and (iii) children in the anxious groups had already visited the location of the research assessment on a previous visit for their clinical assessment. Previous studies have often used greater stress inducing designs by not including the caregiver in the assessment and having a committee of individuals watching the child׳s presentation (e.g., Schmitz et al., 2011). It is possible that in response to tasks that elicit greater anxiety, differences in responding between anxious and nonanxious children become apparent. Future studies might also benefit from monitoring children׳s autonomic arousal in daily stressful situations at school or at home.

Despite being a marginal finding, it is important to mention that nonanxious children showed somewhat greater HR recovery from both tasks compared to anxious children which is in line with previous studies who report significant differences between anxious and nonanxious children (Krämer et al., 2012; Schmitz et al., 2011) and Friedman׳s (2007) autonomic flexibility model. Although any suggestions must be tentative, it is possible that anxious children engage in greater post-event processing after an anxiety provoking task which might lead to prolonged anxiety. Further research is necessary to establish how anxious versus nonanxious children feel during the recovery period in order to gain more insight into what might be driving these differences in HR recovery. In addition, we did not find evidence for specificity of autonomic inflexibility among socially anxious children in comparison to children with other anxiety diagnoses. However, as this is the first study to systematically investigate socially anxious versus other-anxious children in their autonomic activity in response to a social and non-social stressor, replication of this finding is needed.

A notable finding of the current study was that, in all children, with increases in state anxiety HR and RSA reactivity decreased during the cognitive stress task. Previous studies that found blunted HR and RSA responses to and recovery from stressors in children with anxiety disorders, also reported greater subjective anxiety in the anxious versus nonanxious groups which, in light of the findings presented here, might explain these differences (Krämer et al., 2012; Schmitz et al., 2011). An alternative, though speculative, explanation for restricted autonomic responding to stress could be, that children with high levels of state anxiety also perceived the puzzle task to be impossible to complete, and as any effort would be futile, showed little RSA and HR reactivity. It has been proposed that physiological engagement with a task follows an inverted U-shape with increases in task difficulty until task success becomes unattainable (Brehm and Self, 1989; Schmitz et al., 2013). As this is the first study to consider how children׳s subjective anxiety during the task influences their physiological arousal, however, replication of this finding is needed. Future studies would benefit from including assessments of children׳s perceived, and actual levels of task success.

It seems noteworthy that significant associations between state anxiety and HR and RSA reactivity were only apparent in the cognitive stress task. Despite our aim to control for the confounding effects of bodily movements, it is possible that changes in posture (i.e., standing up) in the social stress task, influenced physiological responses in a way that made it difficult to detect the effects of trait and state anxiety. In addition, experimenters noticed that the social stress task often elicited excitement in children in the control group, rather than apprehension, which might have influenced their physiology. Future research should aim to include tasks that do not require changes in posture and record measures of a range of emotions in order to disentangle the potentially confounding factors of the children׳s current emotional state.

On the basis of the tripartite model of anxiety, it seems important to investigate further whether physical arousal has a negative impact on children׳s behavior and cognitions during anxiety provoking tasks. Within treatments for childhood anxiety disorders, the findings presented here reinforce the application of strategies that normalize and explain physiological arousal as part of a stress response. Such cognitive restructuring techniques might be particularly useful in the treatment of social anxiety disorder, as previous studies suggest that specifically children with social anxiety disorder interpret their physical symptoms in a catastrophic fashion (Alkozei et al., 2014). The trend for anxious children to show less HR recovery after the tasks also reinforces the application of techniques that reduce arousal after a stressor (e.g., through relaxation techniques) for some children.

### 4.1 Limitations

This study primarily assessed parasympathetic arousal, however previous studies have suggested that sympathetic arousal may be implicated in the development or maintenance of anxiety disorders in children (Schmitz et al., 2011; Yeragani et al., 2001). In addition, certain psychological states (e.g., perceived success, excitement) may play a role in autonomic arousal during stressful tasks (Schmitz et al., 2013); this has not been taken into consideration in the present study. Finally, it is possible that other confounding variables not considered in the present study influenced the results, including children׳s medical history and medication use, sleep and exercise patterns, as well as possible caffeine intake. Future studies would therefore benefit from including a greater variety of physiological and psychological measures to promote a fuller understanding of the role of the autonomic nervous system in childhood anxiety disorders.

## 5 Conclusion

This study provided no evidence that children with an anxiety disorder differ in their autonomic arousal at baseline or in response to stress in comparison to nonanxious children, and only limited evidence to suggest that they show less autonomic recovery after a stressor. There was some support, however, for the suggestion that state anxiety during tasks influences autonomic activity. While further replication is required, the findings of the current study suggest that children with anxiety disorders may not fundamentally differ from non-anxious children in their cardiac responses to stress.

## Role of funding source

Cathy Creswell was funded by a MRC Clinician Scientist fellowship (G0601874) and Anna Alkozei by a University of Reading PhD Studentship.

## Conflict of interest

The authors declare no conflict of interest.

## Figures and Tables

**Table 1:** Sample characteristics.

	**SA**	**ANX**	**NONANX**	
	*N*=30	*N*=30	*N*=30	
	Mean (SD)	Mean (SD)	Mean (SD)	
Age (years)	9.30 (1.62)	9.40 (1.50)	9.36 (1.40)	*F*(2, 89)=.03
Gender (% female)	53%	53%	53%	*χ*²(1)=1.00
BMI	18.58 (3.57)	17.44 (1.99)	17.85 (3.14)	*F*(2, 78)*=99*
SCAS-c total	39.70 (17.93)^a^	31.23 (18.91)	22.10 (12.52)^a^	*F*(2, 86)=7.98⁎⁎⁎
SCAS-c social phobia	7.38 (3.72)^a, b^	4.51 (3.40)^b^	4.25 (3.07)^a^	*F*(2, 81)=6.88⁎⁎
SCAS-p total	39.84 (17.82)^a, c^	30.44 (11.82)^b, c^	11.36 (6.94)^a, b^	*F*(2, 82)=36.75⁎⁎⁎
SCAS-p social phobia	11.61 (9.72) ^a, c, b^	6.37 (4.16) ^b, c^	3.92 (2.65) ^c^	*F*(2, 80)=10.70⁎⁎⁎

BMI: Body Mass Index; SCAS-c/p: Spence Children׳s Anxiety Scale- child/parent report; ª, ^b^ and ^c^ denote groups that significantly differ.

⁎⁎ *p*<.01.

⁎⁎⁎ *p* <.001.

**Table 2:** Child diagnostic characteristics.

	*n* (%)		
	**SA**	**ANX**	
	*N=*30	*N=*30	
**Primary diagnosis**			
Separation anxiety disorder	0 (.00)	9 (30.00)	
Social anxiety disorder	30 (100.00)	0 (.00)	
Specific phobia	0 (.00)	7 (23.33)	
Agoraphobia w/o panic disorder	0 (.00)	3 (10.00)	
Generalized anxiety disorder	0 (.00)	10 (33.33)	
Anxiety disorder not otherwise specified	0 (.00)	1 (3.33)	
**Overall diagnoses**			
Separation anxiety disorder	14 (46.66)	17 (56.66)	*χ*² (1)=.60
Social anxiety disorder	30 (100.00)	0 (.00)	*χ*² (1)=49.09⁎⁎⁎
Specific phobia	12 (40.00)	15 (33.33)	*χ*²(1)=.61
Panic disorder w/o agoraphobia	1 (3.33)	0 (.00)	*χ*²(1)=1.01
Panic disorder w agoraphobia	1 (3.33)	0 (.00)	*χ*²(1)=1.01
Agoraphobia w/o panic disorder	3 (10.00)	3 (10.00)	*χ*²(1)=.00
Generalized anxiety disorder	17 (56.66)	13 (43.33)	*χ*²(1)=1.06
Obsessive–compulsive disorder	2 (6.66)	0 (.00)	*χ*²(1)=2.06
Anxiety disorder not otherwise specified	0 (.00)	1 (3.33)	*χ*²(1)=1.01
**Comorbid depressive disorder**	3 (10.00)	2 (6.66)	*χ*²(1)=.21
**Comorbid externalizing disorder**	10 (33.33)	4 (13.33)	*χ*²(1)=2.78

⁎⁎⁎ *p* <.001.

**Table 3:** HR and RSA reactivity scores.

	**SA**	**ANX**	**NONANX**	
	(*N*=30)	(*N*=30)	(*N*=30)	
	Mean (SD)	Mean (SD)	Mean (SD)	
**RSA reactivity**				
Social stressor	−.96 (.69)	−.90 (.84)	−1.10 (.64)	*F*(2, 84)=1.57
Cognitive stressor	−.59 (.68)	−.65 (.61)	−.56 (.70)	*F*(2, 84)=.43
**HR reactivity**				
Social stressor	10.98 (9.95)	7.71 (8.61)	11.10 (8.40)	*F*(2, 84)=1.57
Cognitive stressor	5.75 (6.71)	4.76 (6.38)	5.40 (8.63)	*F*(2, 84)=.43
**RSA recovery**				
Social stressor	.90 (.83)	.99 (.76)	1.15 (.71)	*F*(2, 84)=2.62
Cognitive stressor	.45 (.63)	.59 (.63)	.71 (.58)	*F*(2,84)=2.55
**HR recovery**				
Social stressor	−12.18 (8.90)	−10.06 (7.03)^a^	−13.80 (8.09)^a^	*F*(2, 84)=2.62^†^
Cognitive stressor	−3.30 (7.22)^a†^	−3.73 (6.92)^b†^	−6.62 (5.37)^a, b†^	*F*(2, 84)=2.55^†^
**State anxiety**				
Social stress task	4.41 (3.39)	4.00 (3.16)	3.60 (3.60)	*F*(2, 81)=.48
Cognitive stressor	2.12 (2.52)	1.44 (2.00)	1.63 (2.44)	*F*(2, 81)=.57

HR=heart rate; RSA=respiratory sinus arrhythmia; ª and ^b^ denote groups that significantly differ; † marginal difference (*p*<.09); Reactivity and recovery scores are *change* scores from pre-task baseline to task and from task to post-task baseline (Reactivity=Task HR/RSA – Baseline HR/RSA; Recovery=Baseline after the task HR/RSA – Task HR/RSA).
